# *TP53* somatic mutations in Asian breast cancer are associated with subtype-specific effects

**DOI:** 10.1186/s13058-023-01635-2

**Published:** 2023-04-26

**Authors:** Mohana Eswari Ragu, Joanna Mei Ch’wan Lim, Pei-Sze Ng, Cheng-Har Yip, Pathmanathan Rajadurai, Soo-Hwang Teo, Jia-Wern Pan

**Affiliations:** 1 Cancer Research Malaysia, No. 1, Jalan SS12/1A, 47500 Subang Jaya, Malaysia; 2 Subang Jaya Medical Centre, No. 1, Jalan SS12/1A, 47500 Subang Jaya, Malaysia; 3 University Malaya Cancer Research Institute, Faculty of Medicine, University Malaya, Kuala Lumpur, Malaysia; 4 Jeffrey Cheah School of Medicine and Health Sciences, Monash University Malaysia, Subang Jaya, Selangor Malaysia

**Keywords:** TP53, Breast cancer, Asian population

## Abstract

**Background:**

Recent genomics studies of breast cancer in Asian cohorts have found a higher prevalence of *TP53* mutations in Asian breast cancer patients relative to Caucasian patients. However, the effect of *TP53* mutations on Asian breast tumours has not been comprehensively studied.

**Methods:**

Here, we report an analysis of 492 breast cancer samples from the Malaysian Breast Cancer cohort where we examined the impact of *TP53* somatic mutations in relation to PAM50 subtypes by comparing whole exome and transcriptome data from tumours with mutant and wild-type *TP53*.

**Results:**

We found that the magnitude of impact of *TP53* somatic mutations appears to vary between different subtypes. *TP53* somatic mutations were associated with higher HR deficiency scores as well as greater upregulation of gene expression pathways in luminal A and luminal B tumours compared to the basal-like and Her2-enriched subtypes. The only pathways that were consistently dysregulated when comparing tumours with mutant and wild-type *TP53* across different subtypes were the mTORC1 signalling and glycolysis pathways.

**Conclusion:**

These results suggest that therapies that target *TP53* or other downstream pathways may be more effective against luminal A and B tumours in the Asian population.

**Supplementary Information:**

The online version contains supplementary material available at 10.1186/s13058-023-01635-2.

## Introduction

Breast cancer continues to be the most common cancer in Asian women, and the incidence rates of breast cancer among Asian women is predicted to increase in the coming years [36]. Breast cancer in Asian women appears to have a distinct clinical presentation, with a younger age of onset and increased frequencies of HER2 + tumours relative to European populations [29]. Increasingly, genomics studies of breast tumours in Asian women suggest that they have a distinct molecular profile as well, with a more active immune microenvironment and higher frequencies of *TP53* somatic mutations compared to Caucasian women [13, 29, 41, 42]. Interestingly, even though *TP53* somatic mutations are generally more common in ER- tumours, the increased prevalence of *TP53* somatic mutations in Asian women relative to Caucasian women appears to be more pronounced in ER + tumours [2].

The p53 protein encoded by *TP53* is involved in wide range of cellular stress responses, with a wide range of downstream effects including cell cycle arrest, apoptosis, senescence, DNA repair or changes in metabolism [19]. Somatic *TP53* mutations are typically missense mutations that occur in almost every type of human cancer, and are commonly found in the DNA-binding domain of the p53 protein between exons 5 and 8 [26]. The *TP53* mutations possibly instigate whole or fractional loss of protein function or gain of function (GOF) [25, 28].

The frequency and type of TP53 somatic mutations vary across the breast cancer PAM50 molecular subtypes and are most common in the basal-like tumour subtypes and lowest in Luminal A subtype [32]. Early studies in Caucasian populations suggested that the prognostic significance of *TP53* mutations is independent of hormone receptor status [27]; however, more recent studies suggest that *TP53* somatic mutations may have subtype-specific impacts on prognosis [33]. Specifically, mutations in *TP53* were associated with increased mortality in patients with luminal B, HER2-enriched and normal-like tumours but not in patients with luminal A and basal-like tumours [33]. In contrast, *TP53* somatic mutations in Asian breast cancer patients have been associated with slightly better overall survival in ER + patients, but not in ER- patients [29]. Thus, further exploration of the effect of *TP53* somatic mutation in each PAM50 subtype across different populations may uncover population-specific data that could clarify the role of *TP53* as a predictive or prognostic biomarker for breast cancer.

Here we report an analysis of *TP53* somatic mutations in a cohort of 489 Malaysian female breast cancer patients of all ages. In this analysis, we compare various molecular characteristics between tumour samples with mutant *TP53* and wild-type *TP53* across the PAM50 molecular subtypes using whole exome and whole transcriptome data. We identify significant differences in HR deficiency scores, gene expression and molecular pathways that vary between different breast cancer subtypes, but appear to be particularly pronounced in luminal A and luminal B tumours. These results may have clinical implications for the current and future use of therapies that target *TP53* or other downstream pathways in the Asian population.

## Results

### Cohort characteristics

The MyBrCa tumour cohort comprises of 560 female patients of self-reported Malaysian nationality with breast cancer who were recruited sequentially from a single Malaysian private hospital, Subang Jaya Medical Centre (SJMC) [2]. Genetically, this cohort consists of a mix of women with Chinese, Malay or Indian ancestry, but overlaps with other East/Southeast Asian populations according to genotyping analysis [2, 12]. From this initial cohort, we excluded patient samples without RNAseq data as well as patient samples with *TP53* mutations classified as benign, likely benign, or uncertain significance/conflicting interpretations, for a final dataset of 492 samples from 489 patients (3 cases of bilateral tumours. Of the 492 samples, 199 samples were classified as having mutant *TP53* (pathogenic/likely pathogenic somatic mutations), and the remaining 293 samples were classified as wild-type *TP53* to serve as our control group. No samples with germline *TP53* mutations were included in this analysis.

Table 1 shows the relationship between *TP53* somatic mutations and patients’ clinical characteristics, after excluding patients with bilateral tumours. We found significant differences between patients with wild-type vs mutant *TP53* with respect to PAM50 subtypes, tumour grade and histological subtype. Samples with mutant *TP53* were associated with basal-like, Her2-enriched tumours and higher tumour grade, and negatively associated with lobular carcinomas (Table 1). There were no significant differences observed between the two groups with respect to age at cancer diagnosis and tumour stage.

**Table 1:** Cohort characteristics. Statistical significance determined with Student’s t-test or Chi-square tests, excluding “Unknown” samples

Overall (*n*)	Wild-type *TP53* (WT)	Mutant *TP53* (MT)		*p* value (groups)
Subjects [*n*(%)] 486	291	195		
Age at diagnosis (yr) 53.1 ± 11.5	53.3 ± 11.4	52.8 ± 11.7		0.61
*Histological subtype [n(%)]*				
Invasive ductal carcinoma	437	258 (59.0) [88.7]	179 (41.0) [91.8]	0.34
Invasive lobular carcinoma	17	16 (94.1) [5.5]	1 (5.9) [0.5]	3.1E−03
Other	14	5 (35.7) [1.7]	9 (64.3) [4.7]	0.064
Unknown	18	12 (66.7) [4.1]	6 (33.3) [3.1]	
*PAM50 subtypes [n(%)]*				
Luminal A	178	152 (85.4) [52.2]	26 (14.6) [13.3]	< 2E−16
Luminal B	97	67 (69.1) [23.0]	30 (30.9) [15.4]	0.047
HER2-enriched	105	36 (34.3) [12.4]	69 (65.7) [35.4]	3.0E−09
Basal-like	92	30 (32.6) [10.3]	62 (67.4) [31.8]	6.3E−09
Normal-like	14	6 (42.9) [2.1]	8 (57.1) [4.1]	0.19
*Tumour stage [n(%)]*				
0–1	83	51 (61.4) [17.5]	32 (38.6) [16.4]	0.73
2	221	136 (61.5) [46.7]	85 (38.5) 43.6]	0.45
3	147	80 (54.4) [27.5]	67 (45.6) [34.4]	0.11
4	16	12 (75.0) [4.1]	4 (25.0) [2.1]	0.21
Unknown	19	12 (63.2) [4.1]	7 (36.8) [3.6]	
*Tumour grade [n(%)]*				
1	11	11 (100) [3.8]	0 (0) [0]	0.021
2	208	160 (76.9) [55.0]	48 (23.1) [24.7]	6.1E−12
3	232	98 (42.2) [33.7]	134 (57.8) [68.7]	1.0E−13
Unknown	35	22 (62.9) [7.6]	13 (37.1) [6.7]	

Parentheses () represent column percentage and parentheses [] represent row percentage

### Characterisation of TP53 mutations

To investigate the role of *TP53* mutations in the MyBrCa cohort, we began our analysis by comparing the location and distribution of deleterious mutations in the *TP53* gene between the main PAM50 subtypes. The majority of the mutations were substitutions (missense and nonsense mutations (*n* = 139, 69.8%), followed by frame shift indels (*n* = 41, 20.6%) and splice site variants (*n* = 13, 6.5%). However, there was no significant difference in the location of the mutations, with 84.7% of mutations occurring in the DNA binding domain of *TP53* for luminal A and luminal B samples and 86.2% for basal-like and Her2-enriched samples (Additional file 1: Fig. S1). The R273C and R175H mutations, known to be common hotspot mutations in *TP53*, can be observed across all the subtypes at similar frequencies (Additional file 1: Table S1).

### Mutational profiles of tumours with TP53 somatic mutations

**Fig. 1:**
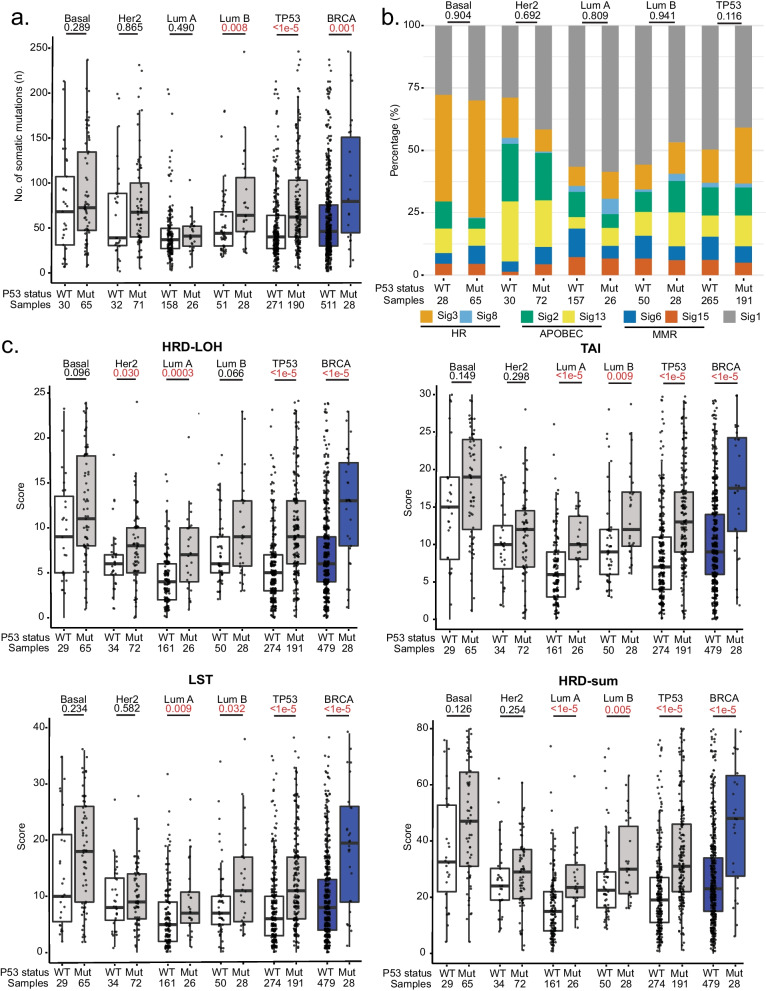
Comparison of mutational profiles for tumours with and without *TP53* somatic mutations. **a** Total number of somatic alterations [single nucleotide variants (SNVs)] and indels identified in mutant and wild-type *TP53* tumours, within each individual PAM50 subtype as well as overall (“TP53”), with germline and somatic *BRCA* mutated tumours (“BRCA”) included for comparison. **b** The stacked bar plot demonstrates the proportion of major mutational signatures in between mutant *TP53* and wild-type TP53 across subtypes. **c** Comparison of genomic scar scores for mutant *TP53* tumours and wild-type *TP53* tumours. Boxplots represent medians (centre line) and interquartile range, and whiskers represent the maximum and minimum values within 1.5 times the interquartile range from the edge of the box. Each data point represents an individual sample. *P* values are derived from Mann–Whitney U tests for figures **a** and **c** and Chi-square tests for figure **b**

Next, we examined the mutational profile of tumour samples for differences associated with mutant *TP53* across subtypes, beginning with an analysis of tumour mutational burden. Using WES data, we established the total number of somatic mutations [small insertion–deletions (indels) and single-nucleotide variants (SNVs)] for each tumour sample and additionally included tumours with known germline and somatic *BRCA* mutations as a positive control. As expected, tumours with germline and somatic *BRCA* mutations had a significantly higher number of somatic mutations compared to non-carriers (*p* = 0.001, Fig. 1a). Similarly, tumours with *TP53* somatic mutations overall had a significantly higher numbers of somatic mutations compared to tumours with wild-type *TP53* (*p* < 1e−5, Fig. 1a). However, although the number of somatic mutations was numerically higher in mutant *TP53* samples compared to wild-type *TP53* samples in each of the breast cancer subtypes, the results were statistically significant only luminal B samples (*p* = 0.008, Fig. 1a).

Following that, we determined the proportion of the major mutational signatures in the tumour samples (Fig. 1b). Mutational signatures are characteristic patterns of mutations associated with different types of DNA damage linked to various exogenous and endogenous mutagens, as well as DNA repair or replicative mechanisms [11]. These signatures, as defined by the COSMIC database (https://cancer.sanger.ac.uk/signatures/), include the age-related signature 1, homologous recombination (HR) deficiency-related signatures 3 and 8, MMR-related signatures 6 and 15 and APOBEC enzyme-related signatures 2 and 13 (Fig. 1b). However, none of these signatures were significantly different between mutant and wild-type *TP53* tumours across the subtypes, suggesting that *TP53* mutations have little effect on the mutational signatures of tumours (Fig. 1b). We did note small increases in the HR deficiency-related signatures 3 and 8 when *TP53* is mutated in all subtypes except Her2-enriched, but these increases were not statistically significant.

We next examined other features of HR deficiency including genomic loss of heterozygosity (LOH), telomeric allelic imbalance (TAI) and large-scale state transition (LST), as well as an overall HRD-sum score that is the total of these scar signature scores [1, 2, 30, 39]. When the results were aggregated across all subtypes, tumours with germline or somatic *BRCA* mutations and tumours with *TP53* somatic mutations had significantly higher HR deficiency scores compared to wild-type tumours (*p* < 1e−5, Fig. 1c). Nevertheless, when examined within subtypes, the increase in scores was more pronounced in luminal A and luminal B tumours and less pronounced in basal-like and Her2-enriched tumours (Fig. 1c). This is reflected in the overall HRD scores (HRD-sum), where, in subtype-specific comparisons, this measure was significantly different between mutant and wild-type *TP53* only in luminal A and luminal B tumours (*p* < 1e−5 and *p* = 0.005), and not in the basal-like (*p* = 0.126) and Her2-enriched (*p* = 0.254) subtypes (Fig. 1c).

Overall, these results suggest that *TP53* somatic mutations are associated with higher numbers of somatic mutations and higher HR deficiency scores, as detectable by whole-exome sequencing. However, these associations appear to be stronger and more consistent in luminal A and B tumours and weaker in Her2-enriched and basal-like tumours, although it is important to note that differences in sample sizes for each subtype may have contributed to this result.

### Differential gene expression analysis

**Fig. 2:**
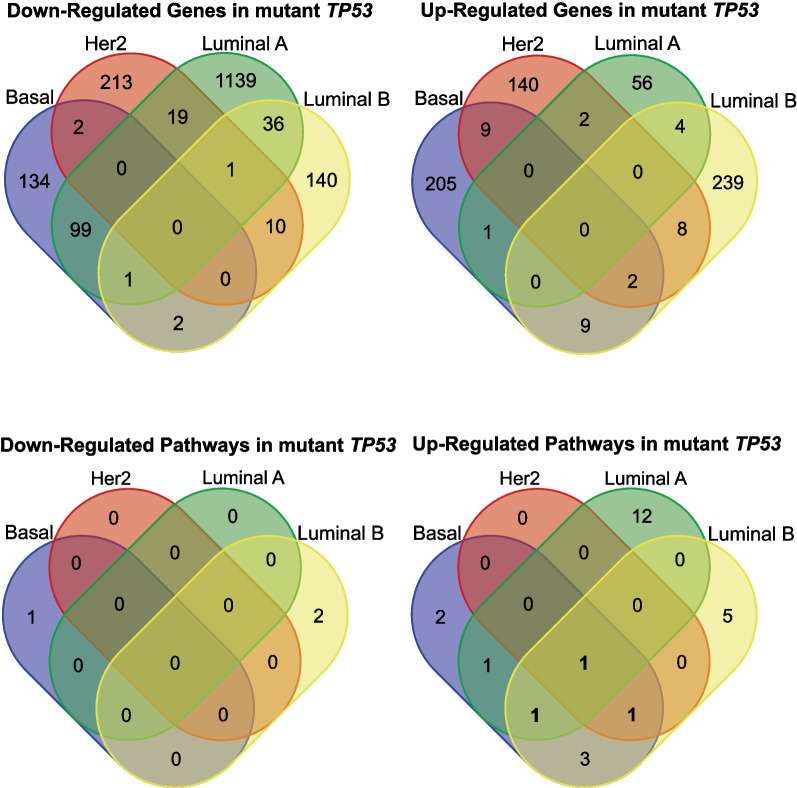
Differentially expressed genes and Hallmark pathways across basal, Her2, luminal A and luminal B subtypes. (Top) The Venn diagrams show downregulated (left) and upregulated (right) genes across the basal, Her2, luminal A and luminal B subtypes. The numbers indicate the number of overlapping differentially expressed genes across subtypes. (Bottom) The lower Venn diagrams display downregulated (left) and upregulated (right) Hallmark gene sets according to gene set enrichment analysis (GSEA) across subtypes. Only genes and Hallmark gene sets with *p* value < 0.05 were included

Next, to determine subtype-specific transcriptomic changes associated with of *TP53* somatic mutations, we employed RNA-Seq data to conduct differential gene expression analyses between tumours with and without *TP53* somatic mutations in each of the four main PAM50 molecular subtypes. The criteria for selecting differentially expressed genes (DEGs) were set as follows: (i) absolute log_2_ fold change more than 1.5; (ii) *p* value less than 0.05. A comparison of upregulated and downregulated DEGs across subtypes revealed that there was remarkably little overlap between different subtypes, with no genes that overlapped across all four molecular subtypes even when the high *p* value threshold of 0.05 was used. Additionally, transcriptomic differences associated with *TP53* somatic mutations appeared to be particularly pronounced in the luminal A subtype, which had a much higher number of significantly downregulated genes compared to the other subtypes (Fig. 2; Additional file 2: Table S2).

To understand this further, we conducted pathway analyses using gene set enrichment analysis (GSEA) [24, 35]. To investigate the overall biological impact of *TP53* somatic mutations on the tumours of MyBrCa patients, we focused on the Hallmark gene sets curated by MSigDB [18]. Comparison of the downregulated and upregulated pathways in tumours with mutant *TP53* revealed only one pathway that was consistently upregulated across all subtypes (mTORC1 signalling), and two pathways that were upregulated in three subtypes (glycolysis and UV response up). Overall, there were 15 Hallmark pathways with significant differences in expression between mutant and wild-type *TP53* in luminal A, 13 in luminal B, 10 in basal-like and only 2 in Her2-enriched. This analysis suggests that the effect of *TP53* mutations is subtype specific, as there were very few overlapping gene sets across molecular subtypes, and the subtypes are observed to have largely distinct upregulated or downregulated pathways when *TP53* is mutated (Fig. 2, Additional file 3: Table S3).

Together, these results suggest that *TP53* somatic mutations are associated with changes to the tumour transcriptome that vary by breast cancer subtype, with surprisingly little overlap. The DEG and GSEA results also suggest that the association between *TP53* somatic mutations and pathway dysregulation is most pronounced in the luminal A subtype and least pronounced in the Her2-enriched subtype.

### Single-sample pathway analysis

To validate and further explore the important pathways associated with mutant *TP53* across subtypes, we performed single-sample GSEA (ssGSEA) analyses. We analysed the ssGSEA results for the hallmark pathways that were observed to overlap across three or four subtypes (mTORC1 signalling, glycolysis and UV response up pathways). We also examined three other Hallmark pathways (P53 pathway, DNA repair and G2M checkpoint) that are associated with known roles of the *TP53* gene such as DNA repair and cell cycle.

**Fig. 3:**
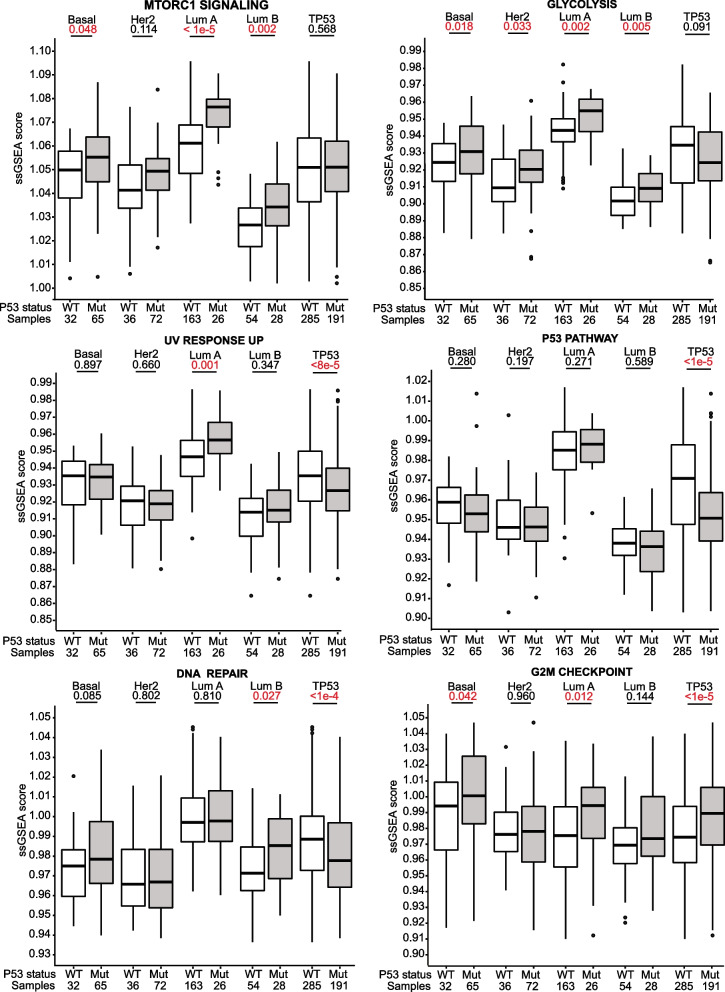
Pathway expression in tumours with and without *TP53* somatic mutations in different breast cancer subtypes. Boxplots compare single-sample GSEA scores for various hallmark pathways in mutant and wild-type *TP53* tumours across different PAM50 subtypes as well as overall (“TP53”). Hallmark pathways were selected if they were previously found to be dysregulated across three or more subtypes in GSEA analysis, or based on known functions of the *TP53* gene. Boxplots represent medians (centre line) and interquartile range, and whiskers represent the maximum and minimum values within 1.5 times the interquartile range from the edge of the box. Each data point represents an individual sample. *P* values are derived from Mann–Whitney U tests

The ssGSEA analyses showed that *TP53* somatic mutations were consistently associated with the mTORC1 signalling and glycolysis pathways across all subtypes, but found that the UV response up pathway was significantly different only in the luminal A subtype (Fig. 3). Additionally, our analyses also showed that the P53 pathway was not significantly dysregulated when comparing mutant and wild-type *TP53* samples within each subtype, while the DNA repair and G2M checkpoint pathways had inconsistent associations with *TP53* somatic mutations (DNA Repair: significantly upregulated only in luminal B (*p* = 0.027), G2M checkpoint: significantly upregulated in luminal A (*p* = 0.012) and basal-like (*p* = 0.042); Fig. 3). Notably, aggregating the data for mutant versus wild-type *TP53* across all subtypes often led to different results in terms of statistical significance and/or the direction of the effect due to the higher prevalence of *TP53* somatic mutations in the basal and Her2-enriched subtypes coupled with differences in gene set expression between the subtypes, emphasising the importance of including tumour subtype as a covariate.

In summary, these data suggest that, across all molecular subtypes, *TP53* somatic mutations are most consistently associated with upregulation of the mTORC1 signalling pathway, as well as the glycolysis pathway to a lesser extent. On the other hand, *TP53* somatic mutations have only small and inconsistent associations with transcriptional alterations in the p53, DNA repair and cell cycle pathways. Similar to our previous results, the differences in pathway expression between samples with mutant and wild-type *TP53* appear to be most pronounced in the luminal A subtype and least pronounced in the Her2-enriched subtype.

### Cancer cell fraction

Next, we hypothesised that subtype-specific differences in the mutational and transcriptional profile of tumours with *TP53* somatic mutations may be due to *TP53* somatic mutations being driver versus passenger mutations in different molecular subtypes. Previous analyses of driver mutations found in tumours from the TCGA dataset have found that most driver mutations are present at a high cancer cell fraction (CCF) [7, 22]. Thus, to assess this hypothesis, we analysed data on the mutational CCF of *TP53* in individual tumour samples that was generated by Pan et al. [29] from copy number data using an ASCAT pipeline. Interestingly, a comparison of *TP53* CCF across breast cancer subtypes showed that the Her2-enriched and basal-like subtypes had lower *TP53* CCFs compared to luminal A and B (Additional file 1: Fig. S2). Furthermore, we also compared samples with high *TP53* CCF to samples with low *TP53* CCF samples (categorised according to the median value) and found that samples with high *TP53* CCF have significantly higher tumour mutational burden and HRD scores in comparison to samples with low *TP53* CCF and wildtype *TP53* samples (Additional file 1: Fig. S3). These findings are consistent with *TP53* mutations acting as driver mutations in luminal A and luminal B tumours but as passenger mutations in the basal-like and Her2-enriched subtypes.

## Discussion

In this study, we compared the molecular profiles of tumours with and without *TP53* somatic mutations in a cohort of 489 Malaysian breast cancer patients. Using whole-exome and transcriptome data, we conducted an analysis of tumour mutational burden, mutational signatures, HR deficiency scores, and differentially expressed genes and pathways. Our analyses resulted in two main findings: (i) the association between *TP53* somatic mutations and HR deficiency scores is stronger in the luminal A and luminal B subtypes, (ii) *TP53* mutations are associated with subtype-specific gene expression differences that are more pronounced in the luminal A and luminal B subtypes, with only the mTORC1 and glycolysis pathways being consistently dysregulated across most subtypes when *TP53* is mutated. We suggest that these results may be due to due to *TP53* somatic mutations generally being driver mutations in luminal A and luminal B tumours as opposed to sometimes being passenger mutations in the basal-like and Her2-enriched subtypes. Indeed, the cancer cell fraction analyses suggest that *TP53* mutations may act as driver mutations in luminal A and luminal B tumours but as passenger mutations in the basal-like and Her2-enriched subtypes. Taken together, these results highlight the importance of considering molecular subtype when examining the role of *TP53* in breast cancer. These results also suggest that therapies for Asian breast cancer that target p53 or other downstream pathways may be more effective in the luminal A and luminal B tumour subtypes.

Given that TP53 is known to have important roles in DNA repair and the maintenance of genomic stability [16, 32], our finding that *TP53* somatic mutations are associated with an increase in tumour mutation burden in some subtypes is not surprising. Similarly, previous research has also shown that *TP53* mutations are associated with chromosomal instability and high HRD scores [4, 15]. Our results confirm that these associations are also present in a large cohort of Asian breast tumour samples.

We also found an association between *TP53* somatic mutations and tumour mutational burden in our data. The direction of causality between *TP53* mutations and tumour mutational burden remains uncertain; a number of the detected *TP53* mutations may be a consequence of increased tumour mutational burden. However, the association between *TP53* mutations with HRD scores, coupled with the lack of association between *TP53* mutations and mutational signatures such as aging or APOBEC-related signatures, suggests to us that, generally speaking, *TP53* mutations affect tumour mutational burden rather than the other way around.

Our results also indicate a strong association between *TP53* somatic mutations and the mTORC1 signalling and glycolysis pathways in Asian breast cancer. The p53 protein is well known to inhibit mTORC1 signalling through multiple mechanisms [6, 8, 10] in response to cellular stresses such as DNA damage. Similarly, *TP53* mutations have been shown to affect energy metabolism at multiple levels in TCGA breast cancer samples and mutant breast cancer cell lines [9, 20].

On the other hand, the weak association between *TP53* somatic mutation and p53 signalling, DNA repair and cell cycle pathways is surprising, given the known functions of TP53. The lack of association between *TP53* mutations and p53 signalling has been noted before in other cohorts [17] and may be due to the existence of compensatory mechanisms [34, 40]. However, other studies have found strong associations between *TP53* somatic mutations and transcriptional changes affecting cell cycle progression [4]. Further research will be necessary to determine if our results can be generalised to the wider Asian population. Notably, when the data for mutant versus wild-type *TP53* are aggregated across all subtypes, the results often lead to misleading conclusions due to differences in sample size, prevalence of *TP53* mutations and expression of various gene sets between various subtypes, highlighting the importance of doing these comparisons in a subtype-specific manner.

Perhaps the most surprising aspect of our results is that the associations between *TP53* somatic mutations and genomic and transcriptomic changes are strongest in the luminal A and B subtypes and weaker in the basal-like and Her2-enriched subtypes. This is surprising because the prevalence of *TP53* somatic mutations is generally lower in the luminal A and B subtypes and higher in the basal-like and Her2-enriched subtypes, such that *TP53* has been considered to be a less important driver gene for luminal A and luminal B subtypes [32]. Our results, on the other hand, suggest that this paradigm may not be as applicable to luminal A and luminal B breast cancers arising in the Asian population, where *TP53* may have a stronger driver role. This may be related to the finding by previous studies that ER + tumours from Asian breast cancer patients have higher frequencies of *TP53* somatic mutations compared to ER + tumours from Western studies, although the mechanism behind this population-specific effect, whether genetic or environmental, remains to be elucidated [13, 29]. Other possible explanations for the lower strength of associations in basal-like and Her2-enriched subtypes are that basal-like tumours are known to have high levels of intra-tumoural heterogeneity, thus diluting the transcriptional effects of sub-clonal *TP53* mutations, while Her2-enriched tumours are primarily driven by *ERBB2* copy number amplification events which renders *TP53* mutations less important.

Limitations of this study include differences in sample sizes for different breast cancer subtypes that may confound our subtype-specific comparisons due to differences in the power to detect significant changes between mutant and wildtype samples. Additionally, our study also did not have a sufficient sample size to account for differences in functional effect across different *TP53* mutations—an important caveat that deserves further analysis in future studies. Accordingly, we have chosen here to limit our study to an aggregate analysis based on a definition of pathogenicity that is similar to what might be encountered in routine clinical use.

In conclusion, *TP53* somatic mutations in Asian breast cancer are associated with HR deficiency and upregulation of the mTORC1 signalling and glycolysis pathways. These associations appear to be stronger in luminal A and luminal B tumours, and weakest in the Her2-enriched subtype, which may be an important consideration for therapies that target *TP53* or other downstream pathways in the Asian population. These results may also provide useful context for further research into *TP53* somatic mutations as predictive or prognostic biomarkers for breast cancers arising in the Asian population.

## Methods

### Whole-exome and transcriptome dataset from the MyBrCa cohort

Our study employed whole-exome and transcriptome data of tumour samples collected from the MyBrCa cohort that has been described previously [29]. The MyBrCa tumour genomics cohort comprised of 560 breast cancer patients recruited as part of the Malaysian Breast Cancer (MyBrCa) study [38] at the Subang Jaya Medical Centre, whose fresh frozen primary tumours were selected to undergo deep whole exome and transcriptome sequencing. Genomics analyses of these patient tumour samples have been approved by the Ethics Committee of Subang Jaya Medical Centre (reference no: 201208.1). More specific details on the sequencing methodology are available from the original publication.

Among the 560 patients, 29 tumour samples that had *TP53* mutations with unknown or uncertain significance or conflicts over its pathogenicity (VUS) were excluded from this study, as only *TP53* somatic mutations that were pathogenic or likely pathogenic according to ClinVar or Varsome were included in this study (see below). Other 39 patients without available WES or RNAseq data were excluded from further analysis for a final sample size of 492 samples. Tumour samples were categorised as either mutant or wild-type *TP53*, and no overlapping samples were included in each set.

### Mapping and variant calling of TP53 mutations

Analysis of sequencing data was performed as described previously in study by Pan et al. [29]. In summary, for WES, sequenced reads were aligned to the human genome b37 plus decoy genome by utilising bwa-mem version 0.7.12 [29]. Local realignment, duplicate removal and base quality recalibration were performed via the Genome Analysis Toolkit (GATK, v3.1.1) [23]. Somatic SNVs were identified via GATK3 Mutect2 [23], whereas small insertions and deletions (indels) were established by Strelka2 [14].

The variants present only in tumour tissue samples were consequently categorised as somatic mutations. The reference sequences for numbering were based on the NCBI GenBank Database for TP53 (NM_000546.6). Clinical variant annotations were obtained from NCBI ClinVar (http://www.ncbi.nlm.nih.gov/clinvar) and Varsome (https://varsome.com/). The variants are considered as pathogenic mutations if they were annotated as “pathogenic” or “likely pathogenic” in either ClinVar or Varsome. *TP53* mutations with unknown or uncertain significance (VUS) or with conflicts over its pathogenicity were excluded from this study. No samples with germline *TP53* mutations were included in the study.

### Mutational signatures

Mutational signatures in each breast tumour sample were determined from annotated VCF files using deconstructSigs [31], using the single base-pair substitution (SBS) signatures described in the COSMIC database.

### HR deficiency scores

The following measures of HR deficiency were established as depicted earlier: [1] LOH, [2] LST and, [3] TAI [2, 39]. Allele-specific copy number (ASCN) profiles on paired normal-tumour BAM files were classified via Sequenza [5] and utilised to analyse the single measure scores and HRD-sum scores via the scarHRD R package [37].

### Gene expression analysis

Gene expression data used for this study were the same as in the original analysis (29). Briefly, RNA-seq reads were mapped to the hs37d5 human genome and the ENSEMBLE GrCh37 release 87 human transcriptome via the STAR aligner (v.2.5.3a) [3]. Variant calling for RNA-seq data was also performed by utilising using the GATK Best Practices workflow for RNA-seq. Gene-level transcript counts were obtained using featureCounts (v. 1.5.3).

### Molecular classification based on gene expression data

Gene-level count matrices for the cohort were transformed into log2 counts-per-million (logCPM) using the voom function from the limma (v. 3.34.9) R package. The transformed matrices were then was subtyped according to PAM50 designations using the Genefu package in R (v. 2.14.0).

### Differential gene expression and functional enrichment analysis

Gene expression was analysed with the DEseq2 package, an R-based open-source software designed to analyse transcriptomic data for differential expression, as previously described [21]. GSEA was then performed for each downregulated and upregulated genes from each PAM50 subtypes using Hallmark Gene set [18, 24, 35]. Enrichment analysis was done with default parameter settings. An enrichment score was calculated for each gene set (KS-statistics) reflecting if the genes in the particular gene set appeared in the top (positive score), in the bottom (negative score), or were randomly distributed (close to zero score). These scores were compared with scores calculated from 1000 randomly permuted gene lists, in order to calculate false discovery rates (FDR) (cutoff at FDR = 0.05). The single sample gene set enrichment analysis (ssGSEA) was applied to analyse the RNA-seq data as well. Hallmark gene sets from the Molecular Signatures Database were used for GSEA and ssGSEA analysis [18, 24, 35].

### Statistical analysis

The Mann–Whitney *U* test and the Chi-square test were executed for comparisons of variables between categories. *P* < 0.05 was considered statistically significant, and all tests were two-sided. Statistical analyses were performed using R v4.0. All box and whiskers plots in the main and Additional file figures are constructed with boxes indicating 25th percentile, median and 75th percentile, and whiskers showing the maximum and minimum values within 1.5 times the inter-quartile range from the edge of the box, with outliers not shown.

## Supplementary Information

**Additional file 1: Fig. S1**. Distribution of somatic *TP53* mutations identified in MyBrCa. **Fig. S2.** Cancer cell fraction (CCF) of *TP53* across somatic mutations. **Table S1. **Common *TP53* somatic mutations in the MyBrCa cohort. **Fig. S3. **Comparison of TP53 mutations carriers with a high TP53 CCF to samples with low TP53 CCF of Tumour Mutational Burden and HRD scores.

**Additional file 2: Table S2.** Differential Gene Expression Analysis across PAM50 subtypes. The table indicates upregulated and downregulated genes in each subtype.

**Additional file 3: Table S3**. GSEA hallmark pathway analysis across PAM50 subtypes. The table indicates the upregulated and downregulated Hallmark pathways in each subtype.

## Data Availability

The data that support the findings of this study are available on the European Genome-phenome Archive under the study accession number EGAS00001004518. Access to controlled patient data will require the approval of the Data Access Committee. Further details and other data that support the findings of this study are available from the corresponding author upon request.
