# Supplementary material for: TP53 somatic mutations in Asian breast cancer are associated with subtype-specific effects
Source: Breast Cancer Res. 2023 Apr 26;25:48. doi: 10.1186/s13058-023-01635-2 (PMC10134541; doi:10.1186/s13058-023-01635-2)
Supplement: Supplementary file 1 — Additional file 1: Fig. S1. Distribution of somatic TP53 mutations identified in MyBrCa. Fig. S2. Cancer cell fraction (CCF) of TP53 across somatic mutations. Table S1. Common TP53 somatic mutations in the MyBrCa cohort. Fig. S3. Comparison of TP53 mutations carriers with a high TP53 CCF to samples with low TP53 CCF of Tumour Mutational Burden and HRD scores. [file 13058_2023_1635_MOESM1_ESM.pdf]

Supplemental Figures & Table

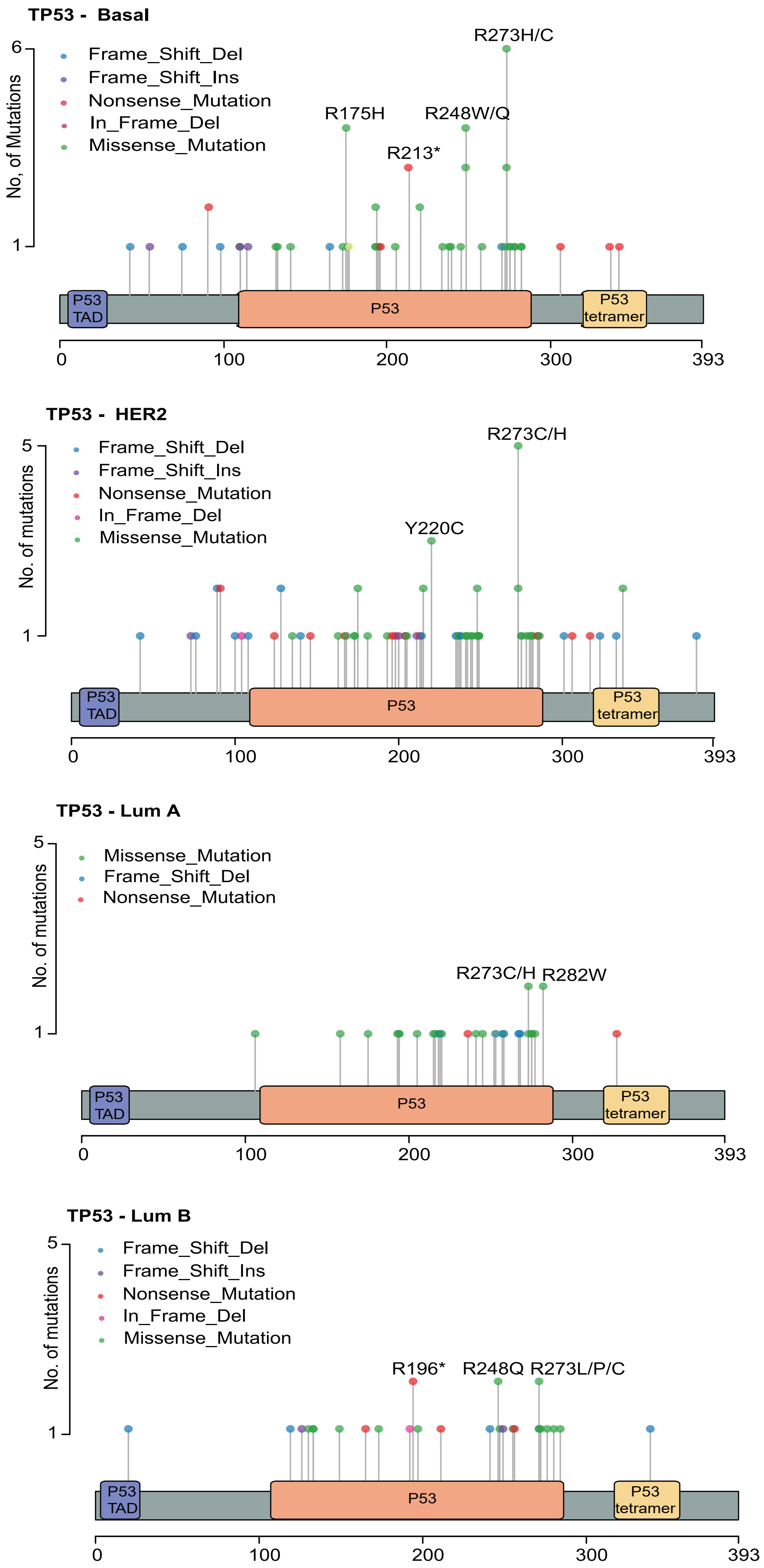

Supp. Figure 1. Distribution of somatic *TP53* mutations identified in MyBrCa. The lollipop plots compare the positions of somatic *TP53* mutations across PAM50 subtypes, with prominent positions indicated.

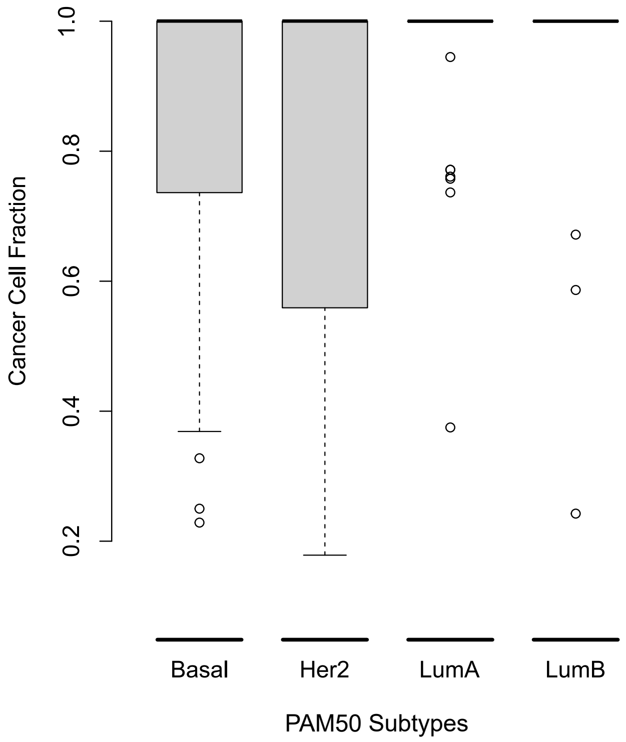

Supp. Figure 2. Cancer Cell Fraction (CCF) of *TP53* across somatic mutations. The proportion of cancer cells carrying somatic mutations in *TP53* across PAM50 subtypes in the MyBrCa cohort

cDNA_Change	Protein_Change	Prevalence (%)				Statistical Significance
		Basal (n=65)	Her2 (n=72)	LumA (n=26)	LumB (n=28)	
**c.524G>A**	p.R175H	6.20	2.80	3.85	3.57	0.797
**c.586C>T**	p.R196*	1.50	1.39	0.00	7.69	0.230
**c.614A>G**	p.Y205C	1.50	1.39	3.85	0.00	0.721
**c.637C>T**	p.R213*	4.62	1.39	0.00	3.57	0.524
**c.659A>G**	p.Y220C	3.07	4.17	3.85	0.00	0.753
**c.743G>A**	p.R248Q	6.15	2.78	0.00	7.14	0.435
**c.817C>T**	p.R273C	4.62	6.95	7.69	3.57	0.857
**c.818G>A**	p.R273H	9.23	2.78	3.85	0.00	0.173

Supp. Table 1. Common *TP53* somatic mutations in the MyBrCa cohort. The table indicates the top ten most common somatic mutations in *TP53* across PAM50 subtypes. The 273C and R175H mutations are common hotspot mutations that can be observed across all subtypes

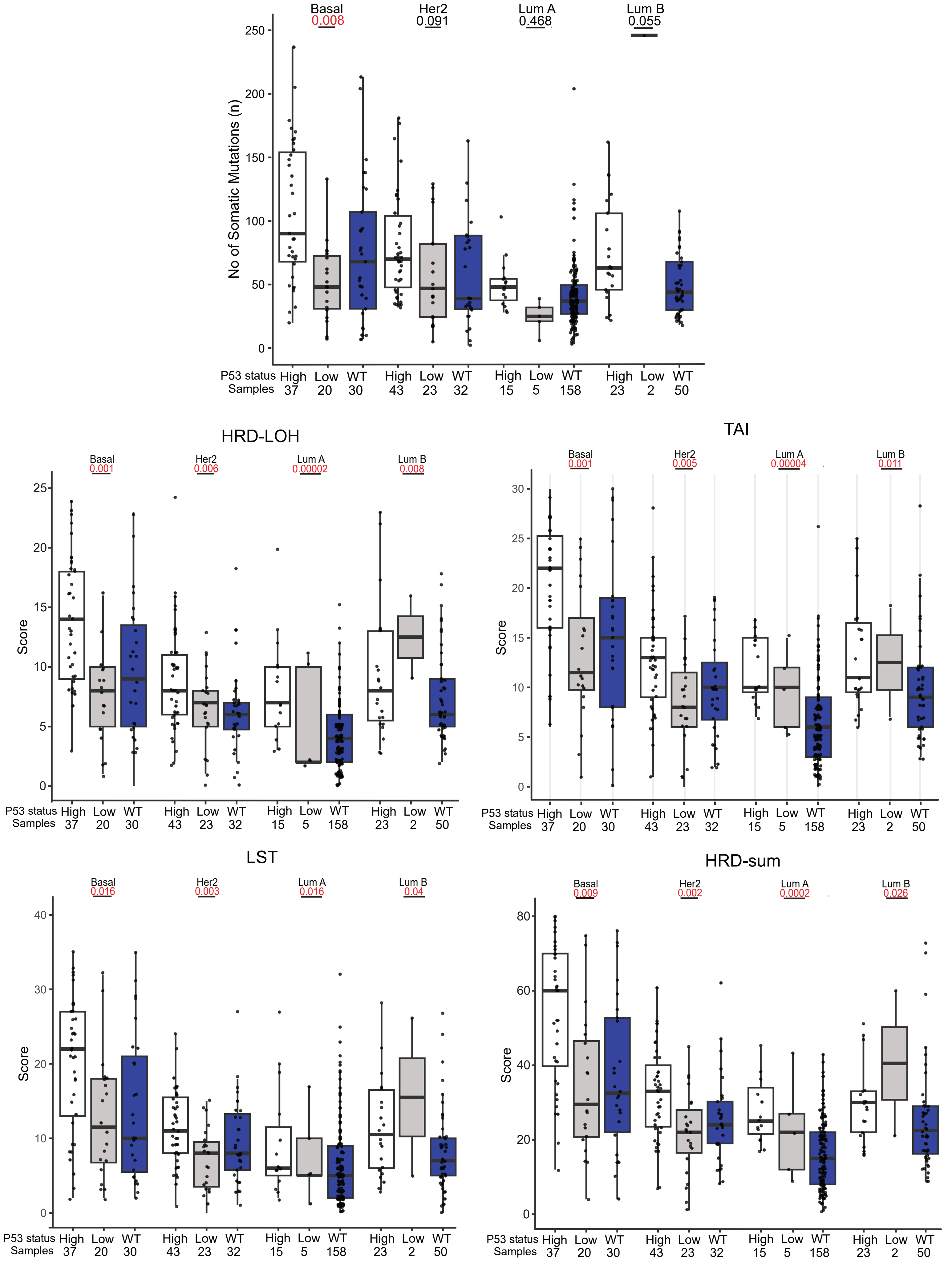

Supp.Figure 3 : Comparison of TP53 mutations carriers with a high TP53 CCF to samples with low TP53 CCF of Tumour Mutational Burden and HRD scores.
